# Dweck’s Growth Mindset Theory in Health Professions Education: A Bibliometric Review

**DOI:** 10.1007/s40670-026-02862-w

**Published:** 2026-09-01

**Authors:** Kelsey D. Frederick, Lisa S. Lewis, Suzanne Larson, Janet Cooley, Anita J. Cleven, Stephanie Dawson, Elizabeth R. Blackwood, Cheryl A. Williams

**Affiliations:** 1 The University of Tennessee Health Science Center College of Pharmacy, 301. S. Perimeter Park Drive, Suite 220, Nashville, TN 37211 USA; 2 Duke University School of Nursing, Durham, NC USA; 3 Midwestern University College of Pharmacy, Glendale, AZ USA; 4 University of Arizona R. Ken Coit College of Pharmacy, Tucson, AZ USA; 5 Pacific University School of Pharmacy, Hillsboro, OR USA; 6 ATI Nursing Education, Leawood, Kansas USA; 7 Duke University Medical Center Library & Archives, Durham, NC USA; 8 Salem State University, Salem, MA USA

**Keywords:** Growth mindset, Dweck, Health professions education, Bibliometric review

## Abstract

Carol Dweck’s Growth Mindset Theory (GMT) has gained increasing attention in health professions education (HPE), yet its development has not been comprehensively mapped. This first bibliometric review analyzed 142 HPE articles identified through a Scopus search and Bibliometrix analysis. Publications increased substantially after 2016, with rapid growth following 2020. Commonly associated concepts included resilience, competency-based education, motivation, and self-regulation. The United States produced the most publications and citations, and medicine, nursing, and pharmacy were the most represented professions. Although GMT scholarship in HPE is expanding, greater collaboration and more robust research designs are needed to strengthen the evidence base.

**Supplementary Information:**

The online version contains supplementary material available at https://doi.org/10.1007/s40670-026-02862-w.

## Introduction

Carol Dweck’s growth mindset theory (GMT) emerged from decades of cognitive science research beginning in the 1970s, exploring why some learners persist in the face of challenges while others disengage [1]. By the 1990s, Dweck formalized these motivational patterns into “implicit theories of intelligence,” distinguishing between beliefs that intelligence and ability are fixed versus malleable [2]. These constructs were later popularized as “fixed” and “growth” mindsets [3, 4].

Dweck’s GMT reflects how individuals perceive intelligence and ability [3, 4]. A fixed mindset views intelligence as fixed, innate, limited, and unchangeable [4], often leading learners to avoid challenges and prioritize performance over learning. Motivated by fear of failure and judgment, a fixed-minded individual may prioritize proving their intelligence rather than improving it [5]. In contrast, a growth mindset views intelligence as malleable and something that can be developed through effort, persistence, feedback, grit, and resilience in the face of challenges or setbacks [2–4]. A growth-minded individual prioritizes developing their intelligence and ability rather than displaying it [3, 4]. Importantly, evidence suggests that mindset itself is malleable and can be cultivated through educational interventions [2–4].

Although GMT has gained considerable attention in educational research, the theory and its application remain subjects of scholarly debate. In particular, questions have been raised regarding the effectiveness of growth mindset interventions in improving academic achievement. A recent systematic review and meta-analysis of 63 studies involving nearly 98,000 students reported substantial methodological concerns across the literature, including weaknesses in study design, analysis, and reporting, as well as potential publication and researcher bias [6]. While a small overall positive effect was initially observed, the effect became nonsignificant after accounting for potential publication bias, and no theoretically meaningful moderators were identified [6]. Furthermore, when analyses were limited to studies demonstrating successful mindset change or to the highest-quality studies, intervention effects on academic achievement were not statistically significant [6]. These findings suggest that the educational benefits of growth mindset interventions may be smaller and more context-dependent than originally proposed and may be more relevant for specific student populations rather than universally applicable [6]. At the same time, proponents argue that growth mindset remains a valuable framework for understanding learner motivation, persistence, and responses to challenge, particularly when integrated with broader educational and psychosocial supports. Consequently, the literature reflects ongoing discussion regarding both the theoretical foundations of GMT and the extent to which mindset interventions produce measurable educational outcomes.

Despite ongoing debate regarding the magnitude and consistency of intervention effects, interest in GMT has grown substantially across educational contexts, including health professions education (HPE).Within HPE, learners routinely encounter high-stakes assessments, rigorous clinical training, performance feedback, and professional identity formation challenges, making constructs such as resilience, adaptability, and responses to failure particularly relevant to educational success and well-being. Systematic and scoping reviews highlight its potential to support resilience, well-being, and professional development, while also identifying gaps and limitations in the literature. For example, a systematic review by Wolcott and colleagues examined nearly 5,000 articles, of which only 14 articles were explicitly grounded in Dweck’s GMT [7]. Most of these articles found congruency with themes such as resilience, well-being, and grit, and the researchers highlighted strategies to cultivate a growth mindset among students and faculty. Similarly, a review of HPE literature by Williams and Lewis noted only 22 of nearly 1,300 articles were distinctly related to Dweck’s GMT and HPE students, and the majority of these works were predominantly non-empirical, highlighting opportunity for empirical and predictive research [8]. A review by Calo and colleagues reported that the combined constructs of grit, resilience, and growth mindset reduced perceived stress among physiology students [9]. In HPE, Richardson and colleagues found that a growth mindset was more effective than a fixed mindset for developing HPE competencies [10]. While these reviews provide valuable insight into applications of GMT in HPE, they do not offer a comprehensive overview of how the landscape of literature developed over time.

Bibliometric analysis enables exploration of publication trends, key contributors, and associated concepts, offering a broader perspective on the evolution of this research area. To date, no bibliometric review has examined GMT in HPE. The present study addresses this gap by analyzing publication patterns, citation impact, key authors and institutions, and associated concepts. The authors aim to map the characteristics and trends in the development of GMT scholarship in HPE and identify priorities for future research, funding, and educational impact.

## Methods

### Data Collection and Eligibility Criteria

**Fig. 1:**
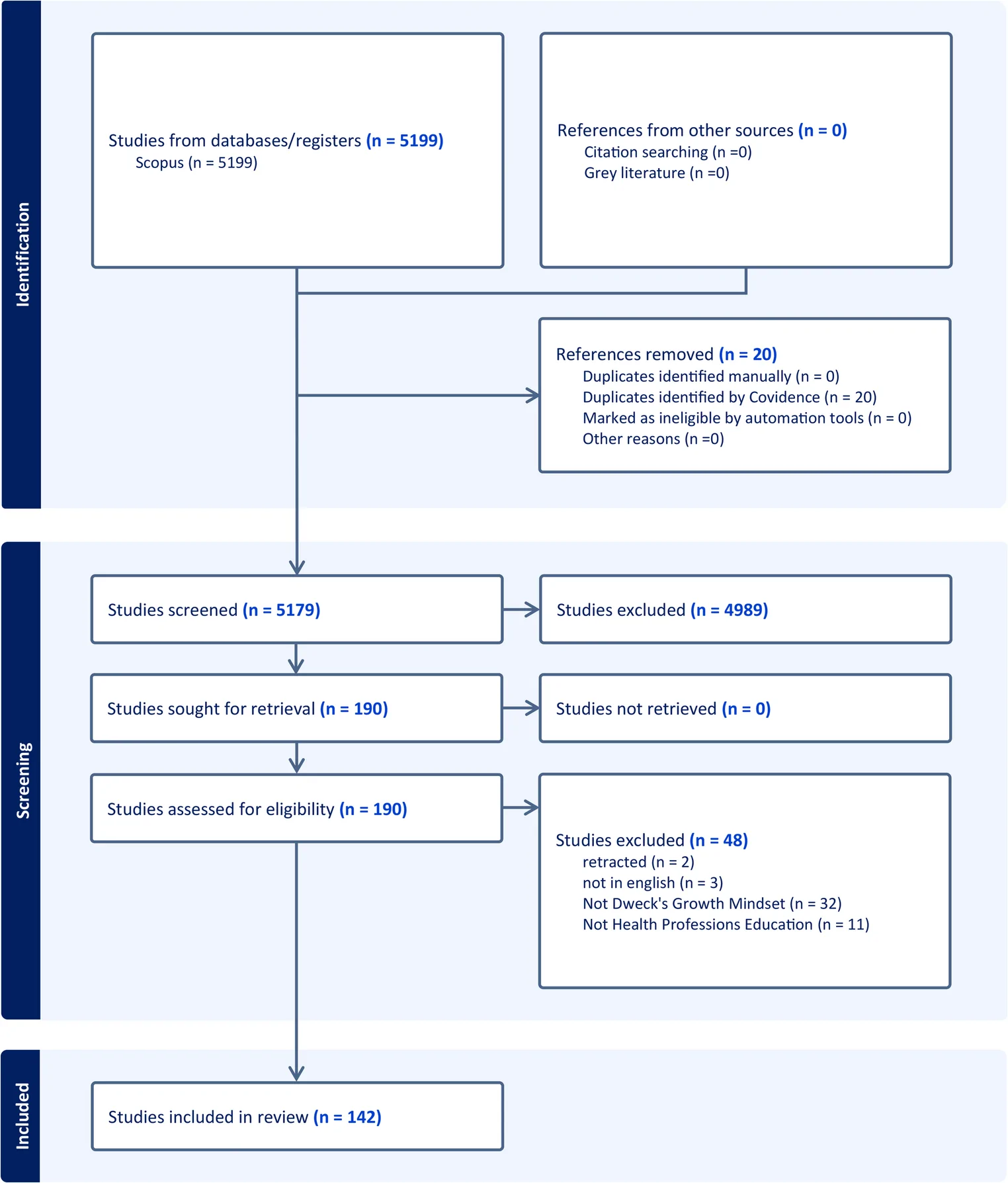
PRISMA flow diagram of study identification, screening, eligibility assessment, and inclusion

Data were collected from the Scopus database (via Elsevier), selected for its comprehensive coverage of high-impact peer-reviewed publications. A professional medical librarian developed the search in consultation with the author team and executed it on June 18, 2025. The search included keywords representing growth mindset, Carol Dweck, and relevant synonyms. The search yielded 5,195 records. Due to the small number of results, no additional filters were applied to maximize capture of relevant literature. The documented search strategy is shown in the PRISMA diagram (Fig. 1). Studies were imported into a Covidence Review for deduplication and screening. Titles and abstracts were independently screened by two members of the author team using predefined eligibility criteria, specifically relating to HPE and Dweck’s GMT. Discrepancies during screening were resolved through discussion among the author team. After screening, 142 articles remained and were included in the review. Scopus was selected because of its broad coverage of peer-reviewed health sciences and educational literature; however, the authors acknowledge that reliance on a single database may have excluded relevant publications indexed elsewhere.

### Data Cleaning

Search results were exported from Covidence, and Scopus unique identifiers (EIDs) were extracted to re-execute the search in the database to collect full bibliographic and citation metadata, which is not retained on Covidence import. Results were then uploaded to the data-cleaning software, OpenRefine, for cleaning and standardization, including consolidation of author names and institutional affiliations. Once cleaned, the author team performed additional data enhancement by categorizing authors (based on affiliation or article topic) to specific health professional fields.

### Data Analysis

The cleaned dataset was analyzed using the R Package Bibliometrix through the Biblioshiny web application, initiated from R Studio. Data extraction from 142 full-text articles was conducted to identify several factors. The type of manuscript and year of publication were considered to observe how much of the literature was evidence-based and whether that changed over time. Professional affiliation, geographic location, and author institution were tracked, as well as if the study or authors were funded. Articles were also analyzed to identify related concepts that were described or studied alongside GMT. In addition to descriptive bibliometric indicators, the analysis explored relationships among authors, institutions, countries, and associated concepts to characterize the intellectual and collaborative structure of GMT scholarship within HPE.

This study followed established reporting guidelines for bibliometric reviews [11].

## Results

**Fig. 2:**
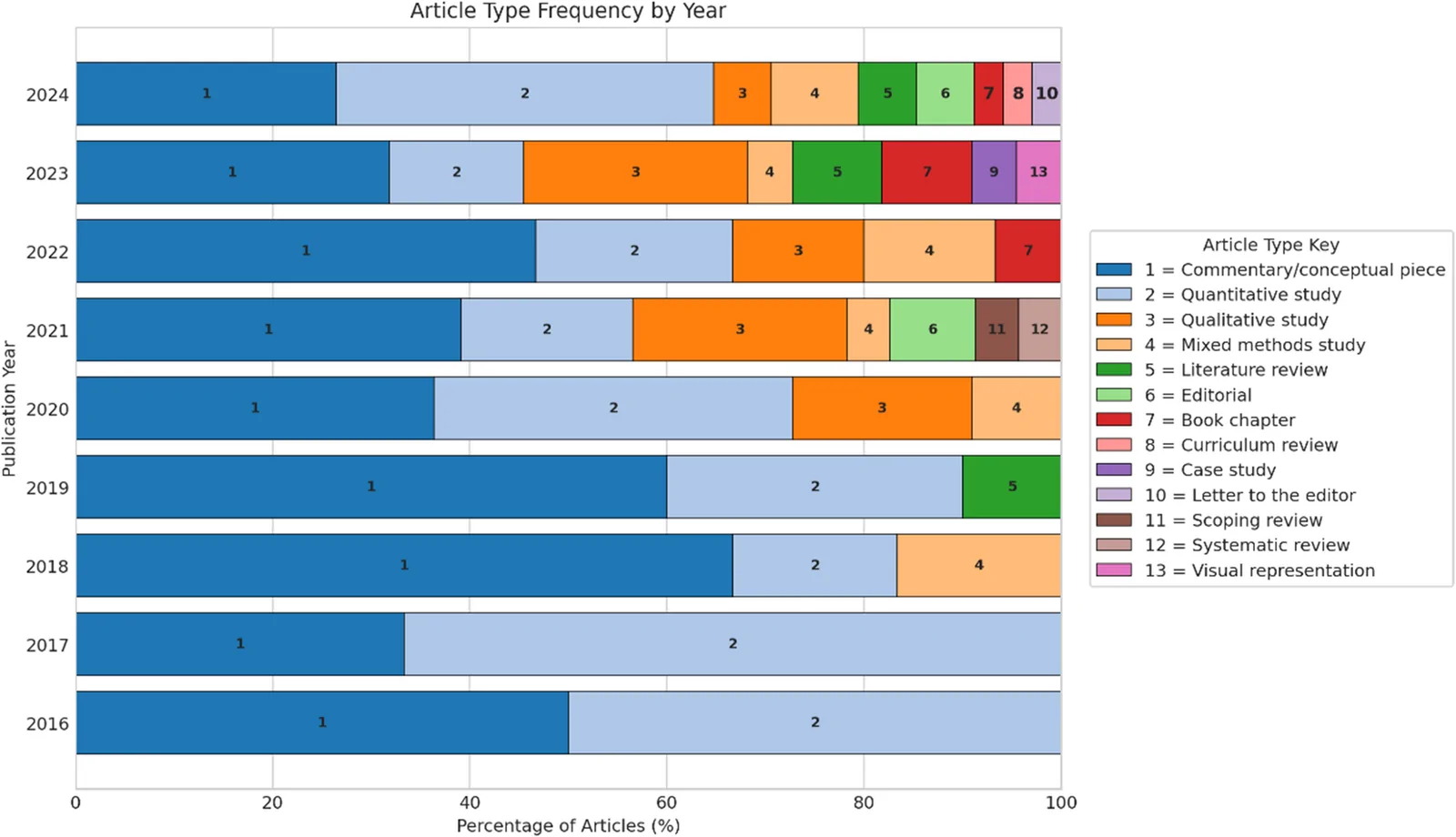
Distribution of article types by publication year

The volume of literature on GMT in HPE has greatly increased over the years (Supplemental File 1). Of the 142 manuscripts included in this review (Supplemental File 2), 49 were commentaries or conceptual articles, 39 were quantitative analyses, 19 were qualitative analyses, 13 were mixed methods studies, 7 were literature reviews, 4 were editorials, and 11 were categorized as other types of manuscripts (e.g., 5 book chapters, 2 case studies, etc.). While commentaries consistently represented a significant portion of the literature per year, there was a clear trend toward increasing research-based articles over time (Fig. 2).

**Fig. 3:**
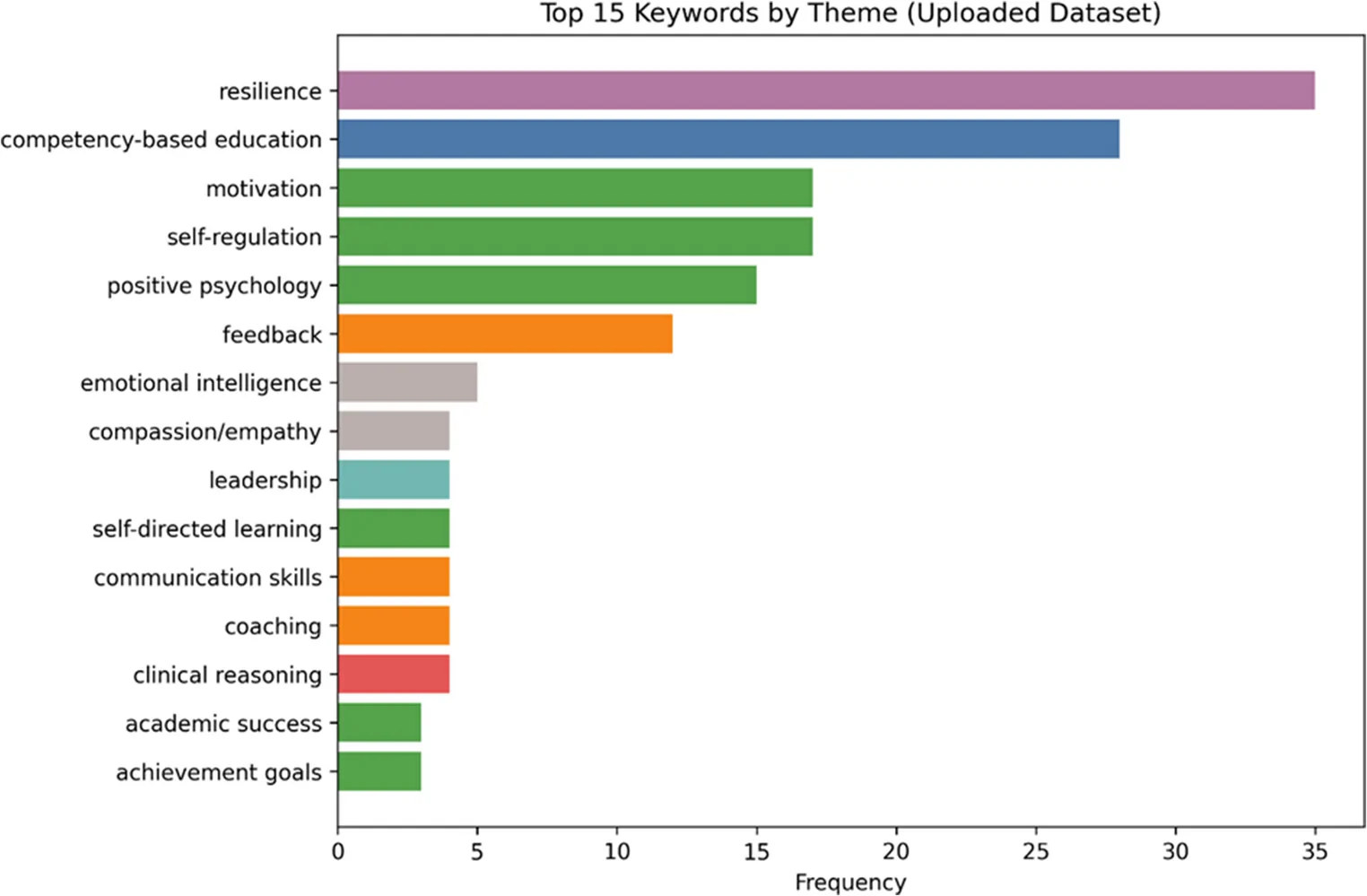
Top 15 keywords associated with growth mindset in the included HPE literature

**Fig. 4:**
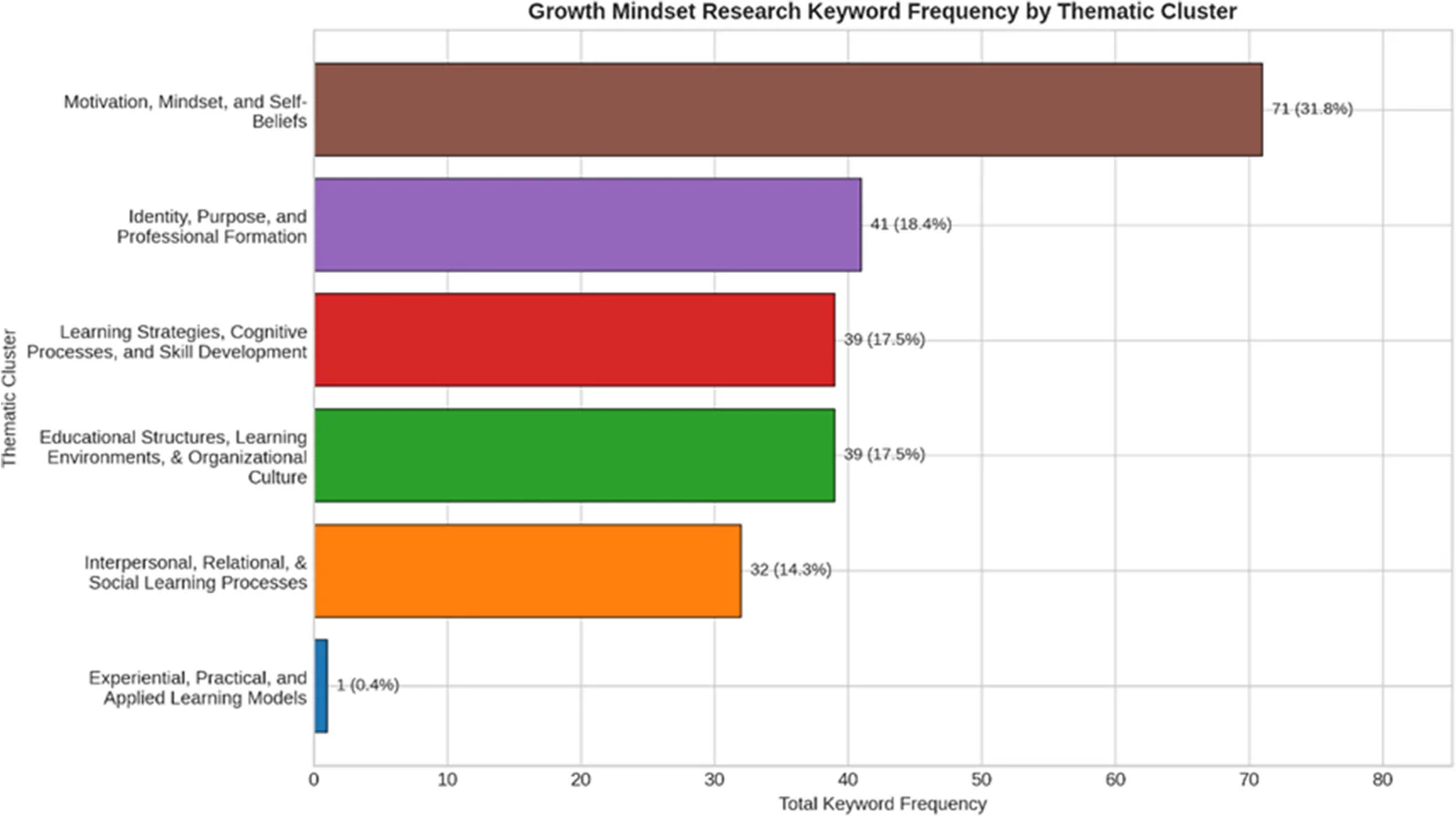
Frequency of growth mindset-related keywords grouped by thematic cluster

More than 60 distinct concepts were described, measured, and assessed in association with growth mindset (Fig. 3). The most common of these were grit/resilience (*N* = 35), competency-based education (*N* = 28), motivation (*N* = 17), self-regulation (*N* = 17), positive psychology/well-being (*N* = 15), and feedback (*N* = 12). Figure 4 clusters keywords by themes. This clustering shows which areas are dense (e.g., learning strategies) versus sparse (e.g., experiential education).

**Fig. 5:**
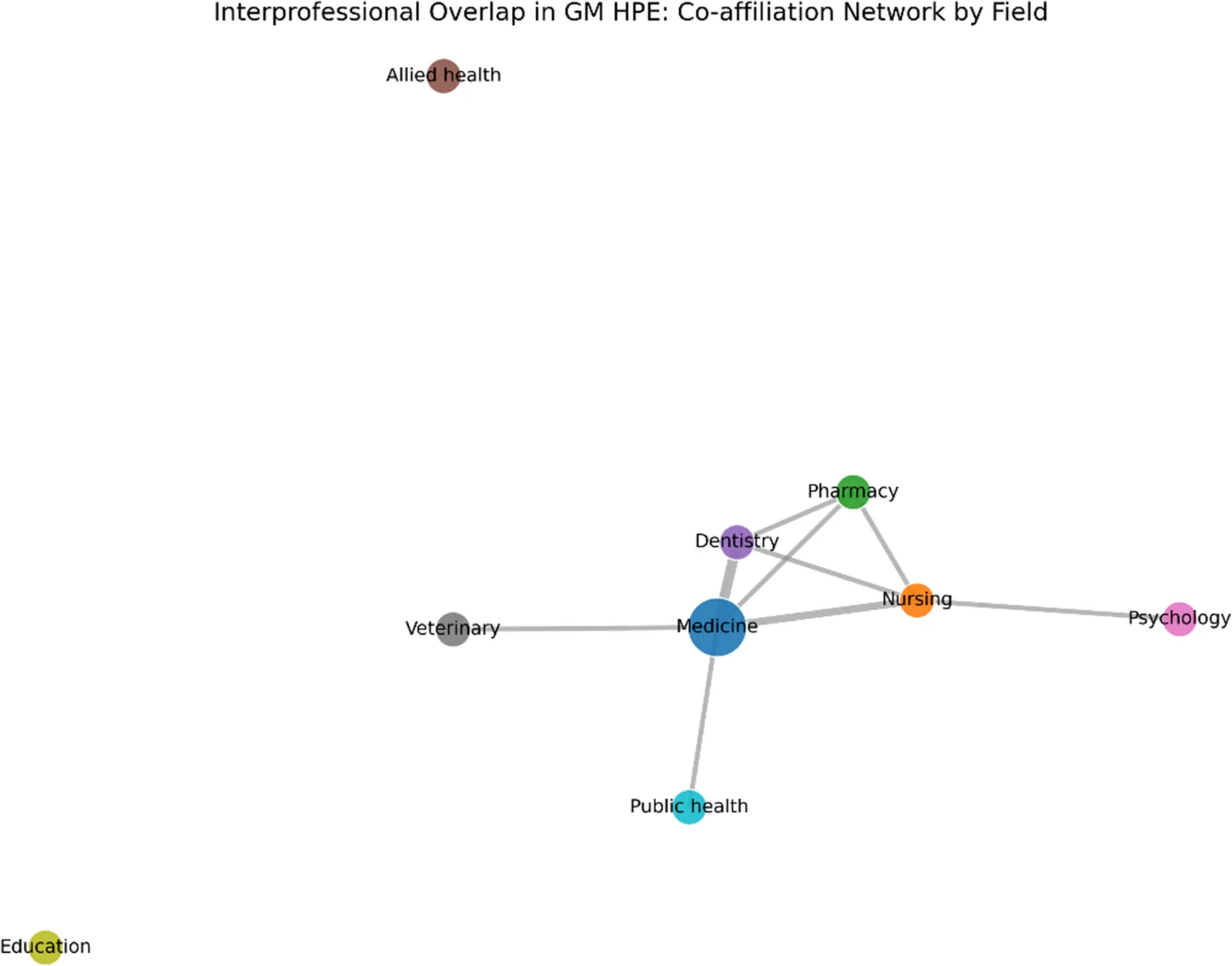
Interprofessional overlap in growth mindset scholarship in HPE. Nodes represent fields (color coded: Medicine = blue; Nursing = orange; Pharmacy = green; Dentistry = purple; Allied health = brown; Public health = teal; Veterinary = gray; Psychology = pink; Education = olive). Node size scales with the number of unique publications (distinct EIDs) attributed to that field; edge width scales with the number of publications listing affiliations that span both fields.

Bibliometric analysis revealed multiple author groups contributed to GMT research; some of these groups have numerous publications. Authors were geographically distributed across the world, with the United States contributing the highest number of publications, followed by Australia and China. Furthermore, authorship networks were largely siloed by discipline (Fig. 5). Regarding citation impact, the United States had the highest total number of citations (*N* = 697; avg article citation 11.4), followed by Canada (*N* = 176; avg article citation 88), Australia (*N* = 96; avg article citation 10.7) and the Netherlands (*N* = 60; avg article citation 60). Additionally, 28 of the manuscripts reported funding support for the project or at least one of the authors.

The three most represented health professions with the highest publication record were medicine, nursing, and pharmacy (Supplemental File 3). The institutions with the highest number of publications on GMT in HPE were Harvard Medical School (*N* = 24), McGill University (*N* = 15), and the University of California (*N* = 10). The top four health professions journals with the greatest number of GMT publications were *Academic Medicine* (*N* = 11), *Medical Education* (*N* = 9), *BMC Medical Education* (*N* = 7), and *Nurse Educator* (*N* = 7).

## Discussion

This bibliometric review provides the first comprehensive mapping of growth mindset scholarship within HPE, revealing both rapid expansion as well as limitations in the field. The findings illustrate a clear trajectory from early conceptual and commentary-driven work toward a growing body of empirical research (Fig. 2), including increased representation of qualitative, quantitative, and mixed methods studies. The increase in GMT articles published aligns with the general increase in research and publications in HPE over recent decades [12] and since the COVID-19 pandemic, which accelerated aspects of academic publishing and prompted an unprecedented rise in how quickly and how much research was released to supply timely evidence for healthcare professionals, policymakers, and the public [13]. As GMT has migrated from psychology and K-12 education to higher and professional education, there has been an increase in debate about its efficacy, scalability, and value for specific populations [14]. This debate may also have contributed to increased examination of the theory.

An important finding emerging from this review is that the growth of GMT scholarship in HPE has occurred alongside increasing debate regarding the effectiveness of growth mindset interventions. While a substantial proportion of the literature describes potential benefits related to resilience, motivation, well-being, self-regulation, and academic persistence, evidence regarding academic achievement outcomes remains mixed. Recent systematic review and meta-analytic evidence has suggested that many growth mindset intervention studies suffer from methodological limitations including weaknesses in study design, analysis, reporting, and potential publication bias [6]. Furthermore, when analyses were restricted to higher-quality studies, effects on academic achievement were generally small or nonsignificant. These findings suggest that growth mindset interventions may not produce universal benefits and that effects may vary according to learner characteristics, educational context, implementation strategies, and outcome measures. Therefore, the expansion of GMT scholarship identified in this bibliometric review should not necessarily be interpreted as confirmation of effectiveness but rather as evidence of sustained scholarly interest in understanding the role of mindset within learning and professional development.

Patterns in authorship and institutional productivity also provide insight into the evolution of the field. The dominance of publications from the United States, along with contributions from Australia, China, and Canada, likely reflects both the global expansion of HPE research and the influence of English-language publication practices. Similarly, the prominence of medicine, nursing, and pharmacy may be shaped by workforce size, academic expectations, and institutional support for scholarship. The U.S. Bureau of Labor Statistics reports 225,360 “health specialties teachers”, the majority of which are working in the university or college setting [15]. This category encompasses dentistry, laboratory technology, medicine, pharmacy, public health, therapy and veterinary medicine. Nursing instructors and teachers are reported separately, and total 72,700, about two-thirds of which are working in the university or college setting [16]. While most academic health professions educators are not tenured or tenure track [17], those working in university and college settings may be encouraged or required to publish for appointment and promotion. Additionally, there has been an overall increase in full-time health professions faculty compared with faculty growth in other academic fields in the past decade [17]. As the largest group of health professions faculty, it is not surprising that nursing faculty were some of the most prolific in publishing on GMT. Given that medical educators are typically employed at doctoral-granting institutions, it is also not surprising that they are the most frequently published.

There is also a pattern of increased output from research teams that receive funding. For example, the Lee Kum Sheung Center for Health and Happiness (at Harvard T.H. Chan School of Public Health) funds studies on positive health, which can include GMT research and the Human Flourishing Program at Harvard’s Institute for Quantitative Social Science studies growth mindset as a component of well-being and resilience. This may explain why Harvard affiliated authors are significant contributors to the GMT literature.

Despite progress, the field remains fragmented, highlighting opportunities to broaden cross-disciplinary participation and interprofessional collaboration. Authorship networks are largely siloed by discipline (Fig. 5), with limited collaboration across professions, institutions, and geographic regions. Many of the challenges in HPE are shared across disciplines, including supporting academic success for marginalized student populations, the widespread advancement of competency-based education, and the need to prepare graduates for the rapidly changing healthcare field and a career of lifelong learning. The GMT itself offers support for navigating many of these challenges, and educators may benefit from shared experiences and conversations about developing growth mindset in learners. Furthermore, collaboration may enhance development of GMT educational interventions, research methods/rigor, and transferability of findings across educational contexts. In addition to collaboration across disciplines, collaboration across institutions and geographic regions may further amplify GMT evidence, impact, and benefits.

The variety of associated concepts identified in this review suggests that researchers frequently study growth mindset alongside resilience, grit, self-regulation, motivation, well-being, feedback, and competency-based education. This observation does not establish causal relationships between these constructs; however, it does indicate that GMT is often positioned as part of broader frameworks of learner development rather than as an isolated educational intervention. It may be that growth mindset is best applied synergistically with other professional development topics and skills, in a dynamic and complementary way. Current evidence often views GMT as an underlying and foundational cognitive and motivational framework that influences motivation, effort, self-regulation, response to feedback and challenges, resilience, teamwork and collaboration, and overall academic success/progress. Future research should move beyond examining associations among these concepts and investigate the mechanisms through which mindset may influence learner outcomes.

Future research should increasingly focus on rigorous evaluation of GMT interventions within HPE. Although the literature has expanded substantially, relatively little is known about which interventions are most effective, under what circumstances they work, and for which learner populations they provide meaningful benefit. Multi-institutional studies using robust experimental designs, longitudinal follow-up, and clinically relevant outcome measures may help address existing concerns regarding methodological quality and strengthen the evidence base for GMT in health professions education.

Finally, this review highlights opportunities for more intentional examination of GMT within HPE curricula. Although mindset is generally regarded as malleable, evidence regarding the effectiveness of specific interventions remains inconsistent. Rather than relying on isolated workshops or brief educational activities, future studies should examine whether sustained curricular approaches that integrate feedback, assessment, reflection, and competency development produce meaningful educational outcomes. Such work may help determine whether GMT can contribute to learner development in ways that are both effective and sustainable.

### Strengths and Limitations

Bibliometric reviews provide several advantages over other types of literature reviews. The present study describes the development and evolution of GMT in HPE over time, identifying types of literature, associated concepts, and patterns of research activity and authorship. This broad view of the literature allows for recognition of the context and gaps related to GMT in HPE.

Several limitations should be considered when interpreting these findings. First, this bibliometric review relied exclusively on the Scopus database. Although Scopus is among the largest multidisciplinary citation databases, grey literature and relevant publications indexed solely in databases such as Web of Science, PubMed, ERIC, or CINAHL may not have been captured. Second, bibliometric analyses are inherently dependent on the accuracy and completeness of indexed metadata. Despite data cleaning and standardization procedures, inconsistencies in author affiliations, institutional naming conventions, and indexing practices may have influenced some findings. Additionally, while bibliometric methods effectively identify publication trends, influential contributors, and research patterns, they do not evaluate methodological quality or the strength of evidence within individual studies. Finally, restricting inclusion based on English language availability may have limited representation and results.

## Conclusion

This bibliometric review represents the first comprehensive mapping of GMT scholarship in HPE, demonstrating substantial growth and diversification of the literature over time. The findings reveal increasing scholarly interest in GMT, particularly in relation to resilience, competency-based education, motivation, self-regulation, well-being, and feedback. At the same time, the literature remains fragmented, with limited collaboration across disciplines, institutions, and geographic regions.

Importantly, this review highlights a gap between the growing volume of GMT scholarship and the strength of evidence supporting educational outcomes. While GMT continues to be explored as a framework for learner development, questions remain regarding the effectiveness, scalability, and long-term impact of mindset interventions in HPE. Future research should move beyond descriptive and correlational studies to include rigorous multi-institutional, longitudinal, and intervention-based research that examines not only academic performance but also outcomes such as clinical reasoning, adaptability, professional identity formation, resilience, and lifelong learning.

Health professions educators may also benefit from greater collaboration across disciplines to develop, evaluate, and refine educational approaches informed by GMT. By strengthening methodological rigor and fostering collaborative scholarship, future research can better determine when, how, and for whom growth mindset approaches contribute meaningfully to learner success. This review provides a foundation for those efforts and identifies key directions for advancing research, educational practice, and theory development in HPE.

## Supplementary Information

Below is the link to the electronic supplementary material.

Supplementary Material 1 (DOCX 86.2 KB)

Supplementary Material 2 (DOCX 46.3 KB)

Supplementary Material 3 (DOCX 98.2 KB)

## Data Availability

Not applicable.
