# Supplementary material for: Dweck’s Growth Mindset Theory in Health Professions Education: A Bibliometric Review
Source: Med Sci Educ. 2026 Sep 1;36(6):3193–201. doi: 10.1007/s40670-026-02862-w (PMC13759675; doi:10.1007/s40670-026-02862-w)
Supplement: Supplementary file 2 [file 40670_2026_2862_MOESM2_ESM.docx]

Supplemental File 2: Articles Included in Bibliometric Review (N=142)

Article Title: Dweck’s Growth Mindset Theory in Health Professions Education: A Bibliometric Review

Journal: Medical Science Educator

Authors:

Kelsey D. Frederick, Pharm.D.

Lisa S. Lewis, EdD, MSN, RN, CNE

Suzanne Larson, Pharm.D.

Janet Cooley, PharmD, BCACP

Anita J. Cleven, PharmD, RPh

Stephanie Dawson, MSN, RN

Elizabeth R. Blackwood, MSLS

Cheryl A. Williams, Ph.D., RN, CNE, ANEF

Corresponding Author:

Kelsey D. Frederick, Pharm.D.

The University of Tennessee Health Science Center College of Pharmacy

301. S. Perimeter Park Drive, Suite 220, Nashville, TN 37211

[kfrederick@uthsc.edu](mailto:kfrederick@uthsc.edu)

Supplemental File 2: Articles Included in Bibliometric Review (N=142)

Alan, A., Ennabe, M., Joshi, N., & Weinand, M. (2023). Emotional intelligence in neurosurgery: Mitigating burnout and enhancing performance. *Surgical Neurology International, 14,* Article SNI_624_2023. https://doi.org/10.25259/SNI_624_2023

Alexander, J. B. (2021). Why evaluate and compare emotional intelligence and improvement mindset of plastic surgery residents. *Journal of Surgical Research, 268*, 748–749. https://doi.org/10.1016/j.jss.2021.06.062

Ang, W. H. D., Chew, H. S. J., Rusli, K. D. B., Ng, W. H. D., Zheng, Z. J., Liaw, S. Y., Ang, N. K. E., & Lau, Y. (2022). Spotlight on noncognitive skills: Views from nursing students and educators. *Nurse Education Today, 117*, Article 105486. https://doi.org/10.1016/j.nedt.2022.105486

Antwertinger, Y. J., Larkin, I., Lau, E. T. L., O’Connor, E. L., & Serrano Santos, J. M. (2020). Transitions to successful careers: Pharmacy, psychology and business students reflecting on practicum feedback. *Professional and Practice-Based Learning, 28*, 47–67. https://doi.org/10.1007/978-3-030-48062-2_3

Armitage-Chan, E., & Maddison, J. (2019). The influences of curriculum area and student background on mindset to learning in the veterinary curriculum: A pilot study. *Veterinary Medicine and Science, 5*(3), 470–482. https://doi.org/10.1002/vms3.174

Atkinson, A., Watling, C. J., & Brand, P. L. P. (2022). Feedback and coaching. *European Journal of Pediatrics, 181*(2), 441–446. https://doi.org/10.1007/s00431-021-04118-8

Babchenko, O., Garland, C. B., Bentz, M. L., & Poore, S. O. (2019). Psychological theory as it applies to surgical training. *Annals of Surgery, 269*(5), 812–814. https://doi.org/10.1097/SLA.0000000000003180

Berg, C., Philipp, R., & Taff, S. D. (2023). Scoping review of critical thinking literature in healthcare education. *Occupational Therapy in Health Care, 37*(1), 18–39. https://doi.org/10.1080/07380577.2021.1879411

Bostock, R., Kinnison, T., & May, S. A. (2018). Mindset and its relationship to anxiety in clinical veterinary students. *Veterinary Record, 183*(20), Article 623. https://doi.org/10.1136/vr.104621

Boyd, T., Harz, D., & Besche, H. (2024). The role of inclusion in collaborative learning. *Medical Science Educator, 34*(4), 909–913. https://doi.org/10.1007/s40670-024-02045-5

Bradley, C. L., Jeter, E., Lee, S., & Cooper, J. B. (2021). Impact of a teamwork and conflict management workshop on growth mindset and team communication. *American Journal of Pharmaceutical Education, 85*(1), Article 8269. https://doi.org/10.5688/AJPE8269

Branfield Day, L., Miles, A., Ginsburg, S., & Melvin, L. (2020). Resident perceptions of assessment and feedback in competency-based medical education: A focus group study. *Academic Medicine, 95*(11), 1712–1717. https://doi.org/10.1097/ACM.0000000000003315

Burger, E., Ghanayem, A., & Mulcahey, M. K. (2024). Promoting leadership development among orthopaedic faculty. *Journal of Bone and Joint Surgery, 106*(15), 1439–1444. https://doi.org/10.2106/JBJS.23.00959

Burns, D., Breckenridge, L. A., Gregory, A., & Nye, C. (2024). Redesigning nurse practitioner clinical education with a Dyad/POD model: A feasibility study. *Journal of the American Association of Nurse Practitioners, 36*(12), 704–710. https://doi.org/10.1097/JXX.0000000000001013

Bynum, W. E., Varpio, L., Lagoo, J., & Teunissen, P. W. (2021). “I’m unworthy of being in this space”: The origins of shame in medical students. *Medical Education, 55*(2), 185–197. https://doi.org/10.1111/medu.14354

Calo, M., Judd, B., Chipchase, L., Blackstock, F., & Peiris, C. L. (2022). Grit, resilience, mindset, and academic success in physical therapist students: A cross-sectional, multicenter study. *Physical Therapy, 102*(6), Article pzac038. https://doi.org/10.1093/ptj/pzac038

Calo, M., Judd, B., & Peiris, C. (2024). Grit, resilience and growth-mindset interventions in health professional students: A systematic review and meta-analysis. *Medical Education, 58*(8), 902–919. https://doi.org/10.1111/medu.15391

Calo, M., Peiris, C., Chipchase, L., Blackstock, F., & Judd, B. (2019). Grit, resilience and mindset in health students. *The Clinical Teacher, 16*(4), 317–322. https://doi.org/10.1111/tct.13056

Cao, M., Zhou, N., Duan, H., Wu, H., Li, F., & Xiang, T. (2025). Growth mindset mediates the effect of core self-evaluation on professional self-efficacy for nursing interns: A cross-sectional study. *Nurse Education Today, 147*, Article 106591. https://doi.org/10.1016/j.nedt.2025.106591

Carlson, E. R., & Tannyhill, R. J. (2020). The growth mindset: A contextualization of faculty development. *Journal of Oral and Maxillofacial Surgery, 78*(1), 1–5. https://doi.org/10.1016/j.joms.2019.07.020

Carraccio, C. (2021). Harnessing the potential futures of CBME here and now. *Academic Medicine, 96*(Suppl. 6), S6–S8. https://doi.org/10.1097/ACM.0000000000004102

Carraccio, C., Lentz, A., & Schumacher, D. J. (2023). Dismantling fixed time, variable outcome education: Abandoning “ready or not, here they come” is overdue. *Perspectives on Medical Education, 12*(1), 68–75. https://doi.org/10.5334/pme.10

Carter, A. N., Evans, J., Seibert, S. A., & Weir, S. (2025). Growth mindset enhancement for new nursing students. *Teaching and Learning in Nursing, 20*(1), e258–e262. https://doi.org/10.1016/j.teln.2024.10.023

Chang, S., Lee, H. Y., Anderson, C., Lewis, K., Chakraverty, D., & Yates, M. (2022). Intervening on impostor phenomenon: Prospective evaluation of a workshop for health science students using a mixed-method design. *BMC Medical Education, 22*(1), Article 824. https://doi.org/10.1186/s12909-022-03824-7

Clark, A. M., & Sousa, B. J. (2018). Definitively unfinished: Why the growth mindset is vital for educators and academic workplaces. *Nurse Education Today, 69*, 26–29. https://doi.org/10.1016/j.nedt.2018.06.029

Cleven, A. J., Renaud, A., Larose-Pierre, M., McQuade, B., Griffin, B. L., Johnson, C., & Hughes, J. A. (2023). Associating growth mindset with emotional intelligence and why it’s needed for professional identity formation. *American Journal of Pharmaceutical Education, 87*(6), Article 100110. https://doi.org/10.1016/j.ajpe.2023.100110

Clifton, M., Millsap, M., Cook, B. I., & Taff, S. D. (2025). Growth mindset: Facilitating resilience and self-efficacy in learners. In M. N. Ikiugu, S. D. Taff, S. Kantartzis, & N. Pollard (Eds.), *Routledge companion to occupational therapy: Theories, concepts and models* (pp. 685–697). Routledge. https://doi.org/10.4324/9781003526766-55

Cole, R., Garrigan, A. G., Peters, S. A., Tilley, L., Conley, S. P., Schwartz, J., Vojta, L., & Rudinsky, S. L. (2023). Medical students’ integration of formative feedback during simulation: A grounded theory study. *Military Medicine, 188*(Suppl. 1), 48–55. https://doi.org/10.1093/milmed/usac378

Cook, D. A., & Artino, A. R. (2016). Motivation to learn: An overview of contemporary theories. *Medical Education, 50*(10), 997–1014. https://doi.org/10.1111/medu.13074

Cooley, J., & Frederick, K. D. (2024). Could coping with your inner critic be the key to unlocking your scholarly writing? *Currents in Pharmacy Teaching and Learning, 16*(6), 392–395. https://doi.org/10.1016/j.cptl.2024.03.013

Cooley, J. H., & Larson, S. (2018). Promoting a growth mindset in pharmacy educators and students. *Currents in Pharmacy Teaching and Learning, 10*(6), 675–679. https://doi.org/10.1016/j.cptl.2018.03.021

Cooper, J. B., Lee, S., Jeter, E., & Bradley, C. L. (2020). Psychometric validation of a growth mindset and team communication tool to measure self-views. *Journal of the American Pharmacists Association, 60*(6), 818–826. https://doi.org/10.1016/j.japh.2020.04.012

Coppersmith, N. A., Esposito, A. C., & Yoo, P. S. (2022). The potential application of mindset theory to surgical education. *Journal of Surgical Education, 79*(4), 845–849. https://doi.org/10.1016/j.jsurg.2022.03.008

Cragun, D., Trepanier, A., Krstić, N., Racobaldo, M., Hunt, P., & Randall Armel, S. (2025). Application of the RIME framework in genetic counseling fieldwork training to assess practice-based competencies. *Journal of Genetic Counseling, 34*(3), Article e2007. https://doi.org/10.1002/jgc4.2007

Cruz Walma, D. A., Cruz Walma, A. M., Khoynezhad, S., Park, S. E., & McKenzie, C. T. (2023). Defining success in healthcare education: US dental student and faculty perspectives. *Journal of Dental Education, 87*(7), 1033–1041. https://doi.org/10.1002/jdd.13217

Dawson, L., & Fox, K. (2018). Can assessment be a barrier to successful professional development? *Physical Therapy Reviews, 23*(1), 1–7. https://doi.org/10.1080/10833196.2017.1336840

Desveaux, L., & Ivers, N. (2024). Practice or perfect? Coaching for a growth mindset to improve the quality of healthcare. *BMJ Quality & Safety, 33*(4), 271–276. https://doi.org/10.1136/bmjqs-2023-016456

Dhaliwal, G., & Hauer, K. E. (2021). Excellence in medical training: Developing talent—not sorting it. *Perspectives on Medical Education, 10*(6), 356–361. https://doi.org/10.1007/s40037-021-00678-5

Dory, V., Danoff, D., Plotnick, L. H., Cummings, B. A., Gomez-Garibello, C., Pal, N. E., & Young, M. (2021). Does educational handover influence subsequent assessment? *Academic Medicine, 96*(1), 118–125. https://doi.org/10.1097/ACM.0000000000003528

Dutt, D. C. S., Razavi, H., & Carr, S. E. (2023). Self-determination theory in ophthalmology education: Factors influencing autonomy, competence and relatedness in medical students. *Medical Education Online, 28*(1), Article 2258633. https://doi.org/10.1080/10872981.2023.2258633

Emmanuel, E. (2021). Get it right: Growth mindset for beginning nursing students. *Nurse Educator, 46*(3), E44. https://doi.org/10.1097/NNE.0000000000000923

Ernst, K., Heisen, C., Tokuyama, M. A., & Bharani, K. L. (2025). Making housestaff feel at home: Impact of workspace interventions on anatomic pathology trainee wellness. *Academic Pathology, 12*(2), Article 100170. https://doi.org/10.1016/j.acpath.2025.100170

Felix, K. J., & Kerluku, J. (2022). Perseverance and grit: Tools for a successful career in healthcare. In M. N. Ikiugu, S. D. Taff, S. Kantartzis, & N. Pollard (Eds.), In *The successful health care professional’s guide* (pp. 23–38). Springer. https://doi.org/10.1007/978-3-030-95950-0_2

Findyartini, A., Ramani, S., McKimm, J., & Fornari, A. (2021). Collaborative writing for clinical educators: Recommendations from a community of scholars. *The Clinical Teacher, 18*(4), 330–335. https://doi.org/10.1111/tct.13329

Furey, M. J., Stemrich, R., Westfall-Snyder, J., Gupta, T., Rapp, M., & Hoffman, R. L. (2025). Can artificial intelligence coach faculty to utilize growth mindset language? A qualitative analysis of feedback statements. *Journal of Surgical Research, 308*, 300–306. https://doi.org/10.1016/j.jss.2025.01.029

Garcia, D., Jedel, I., Rapp‑Ricciardi, M., Lindskär, E., Molander‑Söderholm, K., Fagerström, C., & Sikström, S. (2019). Creative utterances about person‑centered care among future health care professionals are related to reward dependence rather than to a creative personality profile. *Heliyon, 5*(3), Article e01389. https://doi.org/10.1016/j.heliyon.2019.e01389

Garino, A. (2020). Ready, willing and able: A model to explain successful use of feedback. *Advances in Health Sciences Education, 25*(2), 337–361. https://doi.org/10.1007/s10459-019-09924-2

Guttin, T., Light, T. P., & Baillie, S. (2022). Exploring the mindset of veterinary educators for intelligence, clinical reasoning, compassion, and morality. *Journal of Veterinary Medical Education, 49*(5), 603–609. https://doi.org/10.3138/jvme-2021-0057

Guttin, T., McKay, T., Light, T. P., Wise, L. N., & Baillie, S. (2024). Growth mindset in veterinary educators: An international survey. *Journal of Veterinary Medical Education, 51*(2), 248–254. https://doi.org/10.3138/jvme-2022-0128

Hamed, R., & Taff, S. D. (2024). Educating the educator: Applying a growth mindset approach to prepare educators for major changes in occupational therapy education. *American Journal of Occupational Therapy, 78*(6), Article 050776. https://doi.org/10.5014/ajot.2024.050776

Hamner, A., Salguero, R., Nasser, B., & Williams, C. (2025). Embracing the growth mindset: Shifting from pass or fail to pass or practice in practice assessments. *Nurse Educator, 50*(1), E6. https://doi.org/10.1097/NNE.0000000000001703

Harvey, C. J., Leedham‑Green, K. E., Koppel, C., Maini, A., Smith, S. F., Morrell, M. J., & Emerson, M. (2025). Improving medical students’ learning strategies, management of workload and wellbeing: A mixed-methods case study in undergraduate medical education. *BMC Medical Education, 25*(1), Article 71. https://doi.org/10.1186/s12909-025-07118-6

Hauer, K. E., Chang, A., van Schaik, S. M., Lucey, C., Cowell, T., & Teherani, A. (2023). “It’s all about the trust and building a foundation”: Evaluation of a longitudinal medical student coaching program. *Teaching and Learning in Medicine, 35*(5), 550–564. https://doi.org/10.1080/10401334.2022.2111570

Himmelberger, Z. M., Tibbetts, Y., Barron, K. E., Hulleman, C. S., Harootunian, G., & Speicher, M. R. (2024). How can a growth mindset‑supportive learning environment in medical school promote student well‑being? *Families, Systems & Health, 42*(3), 343–354. https://doi.org/10.1037/fsh0000915

Holmboe, E. S., Osman, N. Y., Murphy, C. M., & Kogan, J. R. (2023). The urgency of now: Rethinking and improving assessment practices in medical education programs. *Academic Medicine, 98*(8 Suppl.), S37–S49. https://doi.org/10.1097/ACM.0000000000005251

Hopkins, S. R., Rae, V. I., Smith, S. E., Meldrum, S., & Tallentire, V. R. (2023). From safety net to trampoline: Elevating learning with growth mindset in healthcare simulation. *Advances in Simulation, 8*(1), Article 264. https://doi.org/10.1186/s41077-023-00264-1

Hopkins, S. R., Rae, V. I., Smith, S. E., & Tallentire, V. R. (2024). Trainee growth vs. fixed mindset in clinical learning environments: Enhancing, hindering and Goldilocks factors. *BMC Medical Education, 24*(1), Article 6211. https://doi.org/10.1186/s12909-024-06211-6

Hopkins, S. R., Rae, V. I., & Tallentire, V. R. (2024). Sowing the right seeds: Cultivating a growth mindset for medical admissions. *Academic Medicine, 99*(5), 475. https://doi.org/10.1097/ACM.0000000000005657

Huffman, B. M., Hafferty, F. W., Bhagra, A., Leasure, E. L., & Sawatsky, A. P. (2021). Resident impression management within feedback conversations: A qualitative study. *Medical Education, 55*(2), 266–274. https://doi.org/10.1111/medu.14360

Imanoff, J., Alphonsus, L., & Jackson, J. (2023). Using a growth mindset feedback template to guide written feedback. *Nurse Educator, 48*(6), 315. https://doi.org/10.1097/NNE.0000000000001425

Jamieson, J., Palermo, C., Hay, M., Bacon, R., Lutze, J., & Gibson, S. (2025). An evaluation of programmatic assessment across health professions education using contribution analysis. *Advances in Health Sciences Education*. https://doi.org/10.1007/s10459-025-10444-5

Jamieson, J., & Torre, D. (2025). Twelve tips to afford students agency in programmatic assessment. *Medical Teacher*. https://doi.org/10.1080/0142159X.2025.2459362

Jegathesan, M., Vitberg, Y. M., & Pusic, M. V. (2016). A survey of mindset theories of intelligence and medical error self-reporting among pediatric housestaff and faculty. *BMC Medical Education, 16*(1), Article 574. https://doi.org/10.1186/s12909-016-0574-8

Karunaratne, N., Lyons, K., Molcik, E., & Exintaris, B. (2024). Teamwork baseline assessment tool: Sparking teamwork dialogues in health professions training. *Medical Teacher, 46*(10), 1275–1277. https://doi.org/10.1080/0142159X.2024.2330566

Katz, S., Tang, S. J., Singh, V. A., Abramson, E. L., & Tang, A. J. (2025). “The environment fostered true growth”: Harnessing coaching on an innovative medicine sub‑internship. *Journal of General Internal Medicine, 40*(5), 1018–1022. https://doi.org/10.1007/s11606-024-08928-y

Kenneally, A. M., Schadler, A., & Cain, J. (2023). Fixed mindset and impostor phenomenon: Determining the correlation and prevalence among pharmacy students. *Currents in Pharmacy Teaching and Learning, 15*(1), 62–68. https://doi.org/10.1016/j.cptl.2023.02.009

Khwaja, M. (2023). *Resilience and well‑being for dental professionals*. Wiley. https://doi.org/10.1002/9781119814535

Klein, J., Delany, C., Fischer, M. D., Smallwood, D., & Trumble, S. (2017). A growth mindset approach to preparing trainees for medical error. *BMJ Quality & Safety, 26*(9), 771–774. https://doi.org/10.1136/bmjqs-2016-006416

Kogan, J. R., & Hauer, K. E. (2020). Sparking change: How a shift to Step 1 pass/fail scoring could promote the educational and catalytic effects of assessment in medical education. *Academic Medicine, 95*(9), 1315–1317. https://doi.org/10.1097/ACM.0000000000003515

Kong, J., Xu, X., Xu, J., Han, G., & Xue, Y. (2025). Development of a growth mindset assessment scale for nursing students based on the growth mindset model: A mixed‑method study. *Nurse Education in Practice, 82*, Article 104232. https://doi.org/10.1016/j.nepr.2024.104232

Kuehl, S. E., & Spicer, J. O. (2022). Using entrustable professional activities to better prepare students for their postgraduate medical training: A medical student’s perspective. *Perspectives on Medical Education, 11*(6), 359–364. https://doi.org/10.1007/s40037-022-00731-x

Kustritz, M. V. R. (2017). Pilot study of veterinary student mindset and association with academic performance and perceived stress. *Journal of Veterinary Medical Education, 44*(1), 141–146. https://doi.org/10.3138/jvme.1115-181R1

Lai, B. S., Livings, M. S., D’Amico, M. P., Hayat, M. J., & Williams, J. (2018). A growth mindset pilot intervention for a graduate‑level biostatistics course. *Statistics Education Research Journal, 17*(2), 104–119.

Lane, A. S., & Roberts, C. (2021). Developing open disclosure strategies to medical error using simulation in final‑year medical students: Linking mindset and experiential learning to lifelong reflective practice. *BMJ Simulation & Technology Enhanced Learning, 7*(5), 345–351. https://doi.org/10.1136/bmjstel-2020-000659

Lane, A. S., & Roberts, C. (2022). Contextualised reflective competence: A new learning model promoting reflective practice for clinical training. *BMC Medical Education, 22*(1), Article 3112. https://doi.org/10.1186/s12909-022-03112-4

Levytskyi, A., Vygovska, O., Benzar, I., Golovatiuk, D., & Yaroslavskaya, S. (2024). Growth mindset in medical students and fear of failure. *New Educational Review, 78*(4), 111–123. https://doi.org/10.15804/tner.2024.78.4.08

Lewis, L. S., Williams, C. A., & Dawson, S. (2024). Online mindset training for prelicensure nursing students: A randomized longitudinal study. *SAGE Open Nursing, 10*. https://doi.org/10.1177/23779608241236285

Lewis, L. S., Williams, C. A., & Dawson, S. D. (2020). Growth mindset training and effective learning strategies in community college registered nursing students. *Teaching and Learning in Nursing, 15*(2), 123–127. https://doi.org/10.1016/j.teln.2020.01.006

Lewis, L. S., Williams, C. A., & Dawson, S. D. (2022). Effect of a mindset training intervention on Bachelor of Science nursing students. *Collegian, 29*(4), 471–476. https://doi.org/10.1016/j.colegn.2021.10.016

Liu, T. H., & Sullivan, A. M. (2021). A story half told: A qualitative study of medical students’ self‑directed learning in the clinical setting. *BMC Medical Education, 21*(1), Article 2913. https://doi.org/10.1186/s12909-021-02913-3

Llewellyn, S. (2021). Planting seeds of reflective practice. *Teaching and Learning in Nursing, 16*(4), 375–378. https://doi.org/10.1016/j.teln.2021.06.016

Lombardo, L., Ehlers, J., & Lutz, G. (2023). Mindset and reflection—How to sustainably improve intra‑and interpersonal competences in medical education. *Healthcare, 11*(6), Article 859. https://doi.org/10.3390/healthcare11060859

Memari, M., Gavinski, K., & Norman, M. K. (2024). Beware false growth mindset: Building growth mindset in medical education is essential but complicated. *Academic Medicine, 99*(3), 261–265. https://doi.org/10.1097/ACM.0000000000005448

Messineo, L., Tosto, C., & Allegra, M. (2021). Exploring factors predicting undergraduate healthcare students’ use of learning strategies. *European Journal of Educational Research, 10*(3), 1579–1587. https://doi.org/10.12973/EU-JER.10.3.1579

Mitchell, M., Gray, S. T., & Chen, J. X. (2023). A practical introduction to growth mindsets in surgical training. *JAMA Otolaryngology–Head & Neck Surgery, 149*(7), 569–570. https://doi.org/10.1001/jamaoto.2023.0982

Mohanna, K., Wall, D., Cottrell, E., & Chambers, R. (2023). Constructive feedback. In K. Mohanna, D. Wall, E. Cottrell, & R. Chambers (Eds.), *Teaching made easy: A manual for health professionals* (4th ed., pp. 191–201). CRC Press. https://doi.org/10.1201/9781003352532-18

Mukhtar, M., & Gunderman, R. B. (2022). Instilling a growth mindset in resident orientation. *Academic Radiology, 29*(11), 1763–1764. https://doi.org/10.1016/j.acra.2022.07.009

Murry, L. T., Boyer, J. G., Catledge, K., Gettig, J. P., Travlos, D. V., Zarembski, D., & Kiersma, M. E. (2024). The intersection of growth mindset and accreditation in pharmacy education. *American Journal of Pharmaceutical Education, 88*(6), Article 100711. https://doi.org/10.1016/j.ajpe.2024.100711

Nyanganya, K., Mudzviti, T., & Gavaza, P. (2025). Exploring the link between mindset and psychological wellbeing among pharmacy students in Zimbabwe. *Currents in Pharmacy Teaching and Learning, 17*(4), Article 102300. https://doi.org/10.1016/j.cptl.2025.102300

Ong, A. M. L., & Siau, K. (2022). Value of a growth mindset: Improving endoscopy coaching and mentoring. *Digestive Diseases and Sciences, 67*(3), 753–756. https://doi.org/10.1007/s10620-021-07294-7

Osman, N. Y., & Hirsh, D. A. (2021). The organizational growth mindset: Animating improvement and innovation in medical education. *Medical Education, 55*(4), 416–418. https://doi.org/10.1111/medu.14446

Osman, N. Y., Sloane, D. E., & Hirsh, D. A. (2020). When I say … growth mindset. *Medical Education, 54*(8), 694–695. https://doi.org/10.1111/medu.14168

Ozimek, D., Good, L., Leggieri, A., Morgante, B., Phillips, M., Watson, G., & Wilk, D. (2024). Exploring prelicensure nursing students’ perceptions and attitudes toward mathematics in a concept‑based curriculum. *Teaching and Learning in Nursing, 19*(4), e617–e623. https://doi.org/10.1016/j.teln.2024.05.004

Pal, N. E., Young, M., Danoff, D., Plotnick, L. H., Cummings, B. A., & Gomez‑Garibello, C. (2020). Teachers’ mindsets in medical education: A pilot survey of clinical supervisors. *Medical Teacher, 42*(3), 291–298. https://doi.org/10.1080/0142159X.2019.1679359

Patenaude, B., Hogan, R. E., & Manguvo, A. (2024). Exploring the dynamics of medical students’ exam performance in relation to study habits, growth mindset, confidence levels, and demographics. *Medical Science Educator, 34*(2), 371–378. https://doi.org/10.1007/s40670-024-01976-3

Patterson, D. G., & Yukhymenko‑Lescroart, M. A. (2024). University students’ mindset and effort regulation across the domains of nursing and English. *Psychological Reports*. https://doi.org/10.1177/00332941241232895

Persky, A. M., Medina, M. S., & Castleberry, A. N. (2019). Developing critical thinking skills in pharmacy students. *American Journal of Pharmaceutical Education, 83*(2), 161–170.

Puri, A., Memari, M., Sottile, E. M., Snydman, L. K., Lee, W. W., Bonnema, R. A., Jones, D., & Nandiwada, D. R. (2023). Changing the assessment paradigm: Promoting a growth mindset across the medical education continuum. *American Journal of Medicine, 136*(2), 207–212. https://doi.org/10.1016/j.amjmed.2022.10.004

Quinn, M. A., Grant, L. M., Sampene, E., & Zelenski, A. B. (2020). A curriculum to increase empathy and reduce burnout. *Wisconsin Medical Journal, 119*(4), 258–262.

Rae, V. I., Hopkins, S. R., & Tallentire, V. R. (2024). A call for enriching medical student failure workshops with a growth mindset intervention. *Academic Medicine, 99*(7), 705. https://doi.org/10.1097/ACM.0000000000005724

Rajaragupathy, S., Govindarajan, S., & Ponnusamy, D. (2024). Study of mindset among learners and educators in an Indian medical school—A questionnaire‑based survey. *Journal of Education and Health Promotion, 13*(1), Article 1661. https://doi.org/10.4103/jehp.jehp_1661_22

Ratan, B. M., Love, S. J., Rueda, A. E., Bridges, G. C., Mayer, W. A., Harbott, M. J., & Turner, T. L. (2024). Resident‑as‑teacher: Can it be done with an e‑learning module? *Journal of Graduate Medical Education, 16*(3), 333–338. https://doi.org/10.4300/JGME-D-23-00718.1

Richardson, D., Kinnear, B., Hauer, K. E., Turner, T. L., Warm, E. J., Hall, A. K., Ross, S., Thoma, B., & Van Melle, E. (2021). Growth mindset in competency‑based medical education. *Medical Teacher, 43*(7), 751–757. https://doi.org/10.1080/0142159X.2021.1928036

Ricotta, D. N., Huang, G. C., Hale, A. J., Freed, J. A., & Smith, C. C. (2019). Mindset theory in medical education. *The Clinical Teacher, 16*(2), 159–161. https://doi.org/10.1111/tct.12765

Robinson, K. L., Jackson, B., York, N., & Blair, L. K. (2025). Non‑cognitive factors influencing the development of nursing students’ clinical judgment. *Nurse Educator*. https://doi.org/10.1097/NNE.0000000000001909

Rodriguez, V. (2023). Reimagining clinical teaching tools using a growth mindset. *Radiologic Technology, 95*(2), 138–142.

Routh, J., Paramasivam, S. J., Cockcroft, P., Nadarajah, V. D., & Jeevaratnam, K. (2022). Stakeholder perspectives on veterinary student preparedness for workplace clinical training: A qualitative study. *BMC Veterinary Research, 18*(1), Article 439. https://doi.org/10.1186/s12917-022-03439-6

Sagin, A., Balmer, D., Musheno, R., Olenik, J. M., Dingfield, L., Bennett, N. L., & Dine, C. J. (2024). Lifelong learning indicators in medical students after a novel communication skills session. *Journal of Pain and Symptom Management, 67*(4), e367–e374. https://doi.org/10.1016/j.jpainsymman.2024.01.024

Samora, J. B., Ficke, J. R., Mehta, S., & Weber, K. (2019). True grit in leadership: 2018 AOA critical issues symposium addressing grit, sex inequality, and underrepresented minorities in orthopaedics. *Journal of Bone and Joint Surgery, 101*(10), Article e01276. https://doi.org/10.2106/JBJS.18.01276

Sawicki, J. G., Richards, B. F., Schwartz, A., & Balmer, D. (2023). Measuring the learning orientation fostered by pediatric residency programs with the Graduate Medical Education Learning Environment Inventory instrument. *Academic Pediatrics, 23*(6), 1288–1294. https://doi.org/10.1016/j.acap.2023.03.015

Serebrakian, A. T., Petrusa, E. R., McKinley, S. K., Amador, R. O., Austen, W. G., & Phitayakorn, R. (2021). Evaluating and comparing emotional intelligence and improvement mindset of plastic surgery residents. *Journal of Surgical Research, 268*, 750–756. https://doi.org/10.1016/j.jss.2021.06.061

Setlogelo, B., & Nyoni, C. N. (2024). Grit, academic resilience, and mindset of nursing students: A cross‑sectional study. *International Journal of Nursing Studies Advances, 7*, Article 100253. https://doi.org/10.1016/j.ijnsa.2024.100253

Shapiro, N., & Dembitzer, A. (2019). Faculty development and the growth mindset. *Medical Education, 53*(10), 958–960. https://doi.org/10.1111/medu.13948

Shida, Q. (2024). Mediating effects of achievement goal orientation on the relationship between growth mindset and learning engagement in medical students. *Medicine, 103*(21), e38158. https://doi.org/10.1097/MD.0000000000038158

Shin, Y., Rho, J., Cho, M., Lee, M., Kang, Y. J., & Ihm, J. (2024). Unraveling motives: Identifying the impact of university attendance motives on learning behaviors among dental students. *BMC Psychology, 12*(1), Article 1846. https://doi.org/10.1186/s40359-024-01846-y

Sibicky, S. L., Daugherty, K. K., Chen, A. M. H., Rhoney, D., & Nawarskas, J. (2024). Enabling factors for the implementation of competency‑based curricula in colleges and schools of pharmacy. *American Journal of Pharmaceutical Education, 88*(4), Article 100681. https://doi.org/10.1016/j.ajpe.2024.100681

Smith, A., Barnett, E., & Frates, B. (2022). WholeheartedMD: Medical students embracing vulnerability and finding fulfillment. *American Journal of Lifestyle Medicine, 16*(1), 51–56. https://doi.org/10.1177/15598276211042822

Smith, B. R. (2023). Growth mindset. *AEM Education and Training, 7*(2), e10847. https://doi.org/10.1002/aet2.10847

Solomon, S. R., Atalay, A. J., & Osman, N. Y. (2021). Diversity is not enough: Advancing a framework for antiracism in medical education. *Academic Medicine, 96*(11), 1513–1517. https://doi.org/10.1097/ACM.0000000000004251

Swan Sein, A., Dathatri, S., & Bates, T. A. (2021). Twelve tips on guiding preparation for both high‑stakes exams and long‑term learning. *Medical Teacher, 43*(5), 518–523. https://doi.org/10.1080/0142159X.2020.1828570

Tan, J., Li, H., Zhang, P., & Hirsh, D. A. (2025). Formed in context: A mixed‑methods study of medical students’ mindsets in an Eastern culture. *Medical Education, 59*(2), 210–225. https://doi.org/10.1111/medu.15491

Theard, M. A., Marr, M. C., & Harrison, R. (2021). The growth mindset for changing medical education culture. *EClinicalMedicine, 37*, Article 100972. https://doi.org/10.1016/j.eclinm.2021.100972

Tibbetts, Y., Himmelberger, Z. M., Barron, K. E., Speicher, M. R., & Hulleman, C. S. (2024). Learning mindsets and well‑being and ill‑being among osteopathic medical students. *JAMA Network Open, 7*(6), e2418090. https://doi.org/10.1001/jamanetworkopen.2024.18090

Tison‑Brandon, G., Dickerson, M., Sapetti, L., Johnson, R., & Cianciolo, A. T. (2024). Coachability: A longitudinal curriculum to promote medical students’ growth mindset, feedback utilization, and resilience. *MedEdPORTAL, 20*, 11450. https://doi.org/10.15766/mep_2374-8265.11450

Vojta, L., Roberts, C. L., Cole, R., & Barry, E. S. (2023). Interprofessional experiences of family nurse practitioner students during a military field practicum. *Military Medicine, 188*(Suppl. 1), 56–62. https://doi.org/10.1093/milmed/usad106

Wang, L., Gao, X., Zhang, F., Sun, F., & Wan, G. (2024). Self‑regulated learning strategies adopted by successful Chinese nursing students in the process of learning nursing English. *PLoS ONE, 19*(8), e0308353. https://doi.org/10.1371/journal.pone.0308353

Warm, E. J., Kinnear, B., Lance, S., Schauer, D. P., & Brenner, J. (2022). What behaviors define a good physician? Assessing and communicating about noncognitive skills. *Academic Medicine, 97*(2), 193–199. https://doi.org/10.1097/ACM.0000000000004215

Wash, A., Vogel, S., Tabe, S., Crouch, M., Woodruff, A. L., & Duhon, B. (2021). Longitudinal well‑being measurements in doctor of pharmacy students following a college‑specific intervention. *Currents in Pharmacy Teaching and Learning, 13*(12), 1668–1678. https://doi.org/10.1016/j.cptl.2021.09.032

Whittington, R. E., Rhind, S., Loads, D., & Handel, I. (2017). Exploring the link between mindset and psychological well‑being among veterinary students. *Journal of Veterinary Medical Education, 44*(1), 134–140. https://doi.org/10.3138/jvme.1215-192R

Wicks, M. N. (2024). Are we readying new faculty to use a growth mindset to support student success? *Journal of Nursing Education, 63*(11), 723–724. https://doi.org/10.3928/01484834-20241009-02

Williams, C. (2023). Nursing students’ mindsets and choice of learning strategies. *Nurse Educator, 46*(2), 92–95. https://doi.org/10.1097/NNE.0000000000000870

Williams, C. (2024). Achievement goal theory and mindsets: Keys for competency development in nursing. *Nurse Educator, 49*(5), 274–277. https://doi.org/10.1097/NNE.0000000000001694

Williams, C. A. (2018). Mindsets may matter in nursing education. *Nursing Education Perspectives, 39*(6), 373–374. https://doi.org/10.1097/01.NEP.0000000000000267

Williams, C. A. (2020). Nursing students’ mindsets matter: Cultivating a growth mindset. *Nurse Educator, 45*(5), 252–256. https://doi.org/10.1097/NNE.0000000000000798

Williams, C. A., & Lewis, L. (2021). Mindsets in health professions education: A scoping review. *Nurse Education Today, 100*, Article 104863. https://doi.org/10.1016/j.nedt.2021.104863

Wolcott, M. D., McLaughlin, J. E., Hann, A., Miklavec, A., Beck Dallaghan, G. L., Rhoney, D. H., & Zomorodi, M. (2021). A review to characterise and map the growth mindset theory in health professions education. *Medical Education, 55*(4), 430–440. https://doi.org/10.1111/medu.14381

Xiao, F., Zhang, Z., Zhou, J., Wu, H., Zhang, L., Lin, M., & Hu, L. (2023). The relationship between a growth mindset and the learning engagement of nursing students: A structural equation modeling approach. *Nurse Education in Practice, 73*, Article 103796. https://doi.org/10.1016/j.nepr.2023.103796

Yang, X. J., Gan, Y. T., Wang, Z. J., Wang, J. Y., Duan, X. J., Ma, X., Sun, T., Cao, D. P., & Zhang, S. E. (2025). Does a growth mindset curb anxiety among Chinese medical students? Validation based on propensity score matching. *BMC Psychology, 13*(1), Article 2782. https://doi.org/10.1186/s40359-025-02782-1

Yokoyama, H. (2019). Introducing off‑the‑job training to cardiovascular surgical residency training: A new era of developing competent cardiovascular surgeons. *Surgery Today, 49*(4), 300–310. https://doi.org/10.1007/s00595-018-1747-3

Young, N., & Chang, A. (2024). Creating a positive feedback culture in geriatrics education. In J. J. Christmas & A. N. Hughes (Eds.) *Medical education in geriatrics: Strategies for teaching the care of older adults* (pp. 309–318). Springer. https://doi.org/10.1007/978-3-031-32499-4_21

Zhang, D., Zheng, H., Zhao, Y., Wang, X., Xiao, Q., Zhao, R., Wang, L., Hu, K., Chen, Z., & Zhou, Y. (2024). Unlocking academic achievement of nursing master’s students: Ethical leadership, teacher–student relationship and growth mindset—A cross‑sectional survey study. *BMC Medical Education, 24*(1), Article 6436. https://doi.org/10.1186/s12909-024-06436-5

Zhong, C. S. (2019). Embracing the clunk: Fostering trust by encouraging a “growth mindset.” *Academic Medicine, 94*(5), 616–617. https://doi.org/10.1097/ACM.0000000000002605
