# Supplementary material for: Dweck’s Growth Mindset Theory in Health Professions Education: A Bibliometric Review
Source: Med Sci Educ. 2026 Sep 1;36(6):3193–201. doi: 10.1007/s40670-026-02862-w (PMC13759675; doi:10.1007/s40670-026-02862-w)
Supplement: Supplementary file 3 [file 40670_2026_2862_MOESM3_ESM.docx]

Supplemental File 3: Publications by Healthcare Profession

Article Title: Dweck’s Growth Mindset Theory in Health Professions Education: A Bibliometric Review

Journal: Medical Science Educator

Authors:

Kelsey D. Frederick, Pharm.D.

Lisa S. Lewis, EdD, MSN, RN, CNE

Suzanne Larson, Pharm.D.

Janet Cooley, PharmD, BCACP

Anita J. Cleven, PharmD, RPh

Stephanie Dawson, MSN, RN

Elizabeth R. Blackwood, MSLS

Cheryl A. Williams, Ph.D., RN, CNE, ANEF

Corresponding Author:

Kelsey D. Frederick, Pharm.D.

The University of Tennessee Health Science Center College of Pharmacy

301. S. Perimeter Park Drive, Suite 220, Nashville, TN 37211

[kfrederick@uthsc.edu](mailto:kfrederick@uthsc.edu)
